# The Role of Number Notation: Sign-Value Notation Number Processing is Easier than Place-Value

**DOI:** 10.3389/fpsyg.2012.00463

**Published:** 2012-10-30

**Authors:** Attila Krajcsi, Eszter Szabó

**Affiliations:** ^1^Department of Radiology, Harvard Medical School, Brigham and Women’s HospitalBoston, MA, USA; ^2^Department of Cognitive Psychology, Eötvös Loránd UniversityBudapest, Hungary

**Keywords:** number notation, sign-value notation, place-value notation, comparison, addition, numerical processing

## Abstract

Number notations can influence the way numbers are handled in computations; however, the role of notation itself in mental processing has not been examined directly. From a mathematical point of view, it is believed that place-value number notation systems, such as the Indo-Arabic numbers, are superior to sign-value systems, such as the Roman numbers. However, sign-value notation might have sufficient efficiency; for example, sign-value notations were common in flourishing cultures, such as in ancient Egypt. Herein we compared artificial sign-value and place-value notations in simple numerical tasks. We found that, contrary to the dominant view, sign-value notation can be applied more easily than place-value notation for multi-power comparison and addition tasks. Our results are consistent with the popularity of sign-value notations that prevailed for centuries. To explain the notation effect, we propose a natural multi-power number representation based on the numerical representation of objects.

## Introduction

Numbers can be represented in many ways. Indo-Arabic numbers and Roman numerals are only two well-known examples, while dozens of other notations were invented throughout the history of human culture (Ifrah, 1999; Chrisomalis, 2010). Although the structure of the notation might have an effect on how those numbers are processed, the cognitive properties of this effect are hardly known. More importantly, knowing the finer details of the notational effect might shed light on the mental representation of multi-power natural numbers. The main aim of the present study is to investigate the effect of number notations on simple numerical processes.

In a complex notational system, numbers are decomposed into multiples of powers. For example, a specific number could be denoted as the sum of 1s, 10s, 100s, etc. Different cultures applied different methods to utilize this power decomposition. Many methods used two common structures to denote numbers: sign-value and place-value notations (Table 1). In sign-value notation, powers are denoted by symbols, and the quantity of that power is represented by repeating those symbols. For example, in the Roman notation C, X, and I symbols represent 100, 10, and 1, respectively, and 20 is denoted with the repetition of symbol X twice: XX. Unlike a simple sign-value notation, the Roman notation known today includes also quintuples of the powers of tens, for example “V” as 5 or “L” as 50, and the Roman notation includes subtraction, for example, IV means 5–1. However, some less known notations use the sign-value notation structure more strictly. For example, in the ancient Egyptian hieroglyphic system a stroke (|) was used to denote number 1 and a heel bone (∩) to denote 10. In this notation only the powers of 10 could be symbols, and the numbers were denoted by the sum of these symbols: for example, 23 could be denoted as ∩∩|||. In contrast, in a place-value system, the power is noted by the position in the string, while the quantity is represented by the symbol. For example, in the Indo-Arabic 23, the tens are denoted on the second position from the right, and the quantity of tens is noted by the symbol 2.

**Table 1:** **Number notation in sign-value and place-value notations**.

	**Sign-value notation**	**Place-value notation**
	Roman example: XXIII	Indo-Arabic example: 23
Noting the powers	**Symbol**	**Position**
(e.g., 1, 10, 100 in a base 10 system)	X means ten	□_ (left position) means tens
	I means one	_□ (right position) means ones
Noting the quantity	**Quantity of symbols**	**Symbol**
within a specific	••• Means three	3 Means three
power	•• Means two	2 Means two

Understanding the role of notation in numerical processing relies on at least two factors: first, how multi-power numbers are represented mentally, and second, how the specific notation is transcoded to those mental representations. Regarding the first question, research in recent decades has revealed that humans may represent the very same number in different forms; however, there is no consensus on the nature of multi-digit number representation. In an initial model, McCloskey (1992) proposed an abstract mental structure for multi-digit numbers (e.g., 5070 can be represented as {5} 10EXP3 {7} 10EXP1). In this model, numbers from all notations, such as Arabic numerals or number words, are transcoded to this abstract representation. Alternatively, according to one of the most influential models by Dehaene (1992, 1997), numbers are stored in three different representations: in an imprecise analog magnitude system (i.e., the size of the signal is proportional to the value of the number), in an Arabic visual number form (e.g., 307), and in a verbal form (e.g., 235). While the magnitude system is incapable of storing the exact values of numbers, the Arabic visual number form and verbal representation might handle multi-digit numbers (Dehaene et al., 1993, 1999; Spelke and Tsivkin, 2001).

Turning to the second question, the role of transcoding in handling different number notations has been examined in only a handful of studies. The mental use of the aforementioned abstract multi-power number representation was tested with the utilization of Roman number notation (Gonzalez and Kolers, 1982). Using both Roman and Indo-Arabic notations in numerical tasks simultaneously (e.g., is the V + 3 = VII equation true; is 5 equal to V), it was found that the more Roman numbers were used in a task, the more slowly the participants solved it. Gonzalez and Kolers (1982) originally proposed that the reaction time difference related to different notations reflected notation-dependent representations, which goes against the abstract multi-power number representation model. In contrast, others interpreted the same data that both notations used the same abstract mental number representation and it was the transcoding process that caused the slower response latency in Roman notation (Sokol et al., 1991; McCloskey, 1992). To specify this transcoding process, Noël and Seron (1992) proposed that transcoding Roman numerals to the abstract representation depends on four factors: (a) the first numbers (I, II, and III) work as one-to-one notations and (b) some other symbols (V and X) map numbers directly. These first two processes are fast. On the other hand, other numbers are treated with slower arithmetic tools, as in (c) addition (e.g., VI, VII, and VIII) and (d) subtraction (e.g., IV and IX).

It is not only cognitive scientists who find multi-power representation and number notation important. Mathematicians and historians also provide relevant considerations; however, statements about the efficiency of sign-value vs. place-value notations seem somewhat paradoxical. On one hand, there is a strong consensus in the literature that the place-value system is highly efficient (Cajori, 1924; Menninger, 1992; Zhang and Norman, 1995; Dehaene, 1997; Ifrah, 1999), and numerical tasks can be solved only with serious difficulty in a sign-value system, such as with Roman numbers. However, at least two considerations oppose that bold statement. First, sign-value systems were popular in many cultures even when alternative place-value notation was present (Ifrah, 1999; Chrisomalis, 2010). Second, some less frequently cited theoretical studies suggest that sign-value notations can be easily applied for mathematical purposes, sometimes proposing an even easier method for calculations than the methods applied with place-value systems (Anderson, 1956; Lazarides, 1970; Detlefsen et al., 1976; Kennedy, 1981; Schlimm and Neth, 2008). Thus, some historical and computational considerations question the superiority of place-value notation in all computations. One might ask, if Indo-Arabic numbers are so efficient, why did people not recognize their advantages for such a long time? Is the early popularity of sign-value systems simply an accident, or does it reflect a preference for the representation of numbers in the human mind?

To summarize, the literature on number notations has diverse findings. While we have solid knowledge of multi-power number representation (Dehaene, 1992, 1997; McCloskey, 1992; Dehaene et al., 1999; Spelke and Tsivkin, 2001), and some initial results about some specific notations (Gonzalez and Kolers, 1982; Sokol et al., 1991; McCloskey, 1992; Noël and Seron, 1992, 1997), the influence of number notation on mental numerical processing is hardly understood. Furthermore, while most mathematicians and historians suppose that place-value notations are more efficient than sign-value notations (Cajori, 1924; Menninger, 1992; Zhang and Norman, 1995; Dehaene, 1997; Ifrah, 1999), some other theorists emphasize the efficiency with which sign-value systems could be used (Anderson, 1956; Lazarides, 1970; Detlefsen et al., 1976; Kennedy, 1981; Schlimm and Neth, 2008). Thus, it is difficult to conclude how number notation influences number processing.

In the present study, we investigated the cognitive effect of number notations on mental number processing. More specifically, based on historical considerations (i.e., sign-value numbers were popular even when place-value alternatives were available) and computational considerations (e.g., Roman numbers can be used easily to make calculations), we addressed the simple but fundamental problem of whether place-value notation is more complex for human numerical processing, or if, in contrast, it is the sign-value notation that is more complex for human calculation as suggested by the majority of cognitive and mathematical literature (Cajori, 1924; Menninger, 1992; Zhang and Norman, 1995; Dehaene, 1997; Ifrah, 1999). It is difficult to build on former experiments while exploring this issue. Although a few studies have investigated Roman notation (Gonzalez and Kolers, 1982; Sokol et al., 1991; McCloskey, 1992; Noël and Seron, 1992, 1997; Duyck et al., 2008), they rarely explored the role of number notations *per se*. From the viewpoint of the present study, based on these reports, it is difficult to contrast sign-value and place-value notation processing. First, most of these studies used a paradigm in which Indo-Arabic and Roman numerals were used concurrently in the same trial, such as addition tasks with mixed notations (V + 3 = VII), transcription tasks (is V equal to 4), or processing Roman numbers with Indo-Arabic distracters. Thus, any measured effects might be caused by processing either one notation or the other. Second, if our goal is to compare number notations, then the structure and rules of those notations should be matched as strictly as possible. The Roman numbers and Indo-Arabic numbers that were used in those studies are not matched in this sense because (a) while Indo-Arabic numbers use only the power of the base number (i.e., 1, 10, 100, etc.), Roman numerals use both the power of the base number and quintuple of the powers (i.e., 5, 50, 500, etc.), and (b) Roman notation includes subtraction which might make the notation shorter and thus more readable for experts (Kaufman et al., 1949; Mandler and Shebo, 1982), but at the same time makes Roman notation more complex. Finally, (c) the symbols used in a notational system and the length of the numbers were not controlled in those studies. Third, different representations might be used in different stages of development and expertise (see some specific examples in Gelman and Gallistel, 1978; Siegler, 1999; Delazer et al., 2003), and comparing a well-known place-value system (i.e., Indo-Arabic system) with a less-practiced sign-value system (i.e., Roman numbers) is suboptimal. Thus, the knowledge collected regarding the multi-power number representation and the transcoding processes translating number notations to those representations provide no straight predictions regarding the effects of sign-value and place-value number notations on numerical processing.

To overcome the methodological issues described above, two structurally comparable artificial number notations were designed, and participants solved simple multi-power comparison and addition tasks in the new notations. We hypothesized that, contrary to the conventional view, sign-value notation might be more appropriate for number processing than place-value notation. This hypothesis is based on the relative popularity of sign-value notation in the history of culture (Menninger, 1992; Ifrah, 1999; Chrisomalis, 2010) and on efficient sign-value notation algorithms (Anderson, 1956; Lazarides, 1970; Detlefsen et al., 1976; Kennedy, 1981; Schlimm and Neth, 2008).

## Experiment 1

In the first experiment, participants compared numbers in a new artificial number system. A comparison task was chosen as one of the simplest numerical tasks that also had high importance in ancient cultures (Ifrah, 1999).

### Methods

#### Artificial number notations

**Figure 1:**
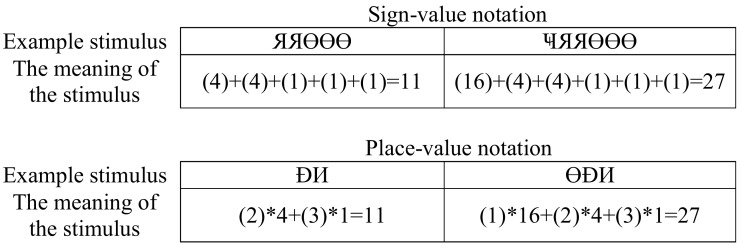
**Examples of numbers in the new notational systems**. These specific examples show the notation of 11 (left), which consists of 2 fours and 3 ones in a base 4 system, and 27 (right), which consists of 1 sixteen, 2 fours, and 3 ones. The correct interpretation of the numbers is visible in the meaning row (participants could not see this explanation). Numbers in parenthesis represent the meanings of the symbols.

To mitigate potentially unavoidable interference with the well-known Indo-Arabic numbers, the new notational systems were as different from our usual notations as possible. Base 4 systems with new characters were designed. All characters had similar vertical and horizontal extent and position, and all of them were visually complex: 0-Ł, 1-Ɵ, 2-Đ, 3-И, 4-Я, and 16-Ҹ (Figure 1).

The two notational systems should differ only in their structure (sign-value vs. place-value), but not in other aspects. First, in contrast with some Roman numbers, such as IV or IX, the sign-value notational system used here did not include subtraction, because subtraction is a deviation from the simplest form of sign-value notation, and it is also a deviation from the place-value structure applied here. Second, the number of symbols a user learns and the length of the numbers should be approximately equal in the two notations. The number of different symbols and the length of the numbers are determined by the base of the number system and the largest power that can be expressed in the task (see Table 2 for a short summary of the relationship between these factors). The number of symbols a user should learn can be equal in the two number notations if the base of the system and the largest power expressed in the numbers are equal (e.g., base three system with the largest number being 3^3^−1, i.e., 26). The length of the numbers in the two notations are closer to each other if the base number and the largest power are small, although it is difficult to perfectly control this aspect of the number systems. Considering these viewpoints, in the present experiment, the base 4 system and three powers were chosen. Thus, the number of symbols was 3 in the sign-value system and 4 in the place-value system. The average length of a number was 4.57 digits in the sign-value notation, and 2.71 in the place-value system.

**Table 2:** **The number of symbols and the length of the numbers depend on the base numbers and the largest expressed powers in sign-value and place-value numbers**.

	Sign-value system	Place-value system
Number of symbols	Largest power: e.g., in a base 10 system, if the largest power is hundred, i.e., 10^3^, three different symbols are used	Base of the system: e.g., the digits from 0 to 9 in base 10
Length of a number	Determined by the base and the power together: e.g., for all powers, within the power, the length can be between 0 and the base number	Largest power: e.g., in a base 10 system if the largest power is hundred, i.e., 10^2^, then the longest number can consist of three digits

#### Incorrect strategies in the comparison task

The comparison tasks can be solved by incorrect strategies. For example, one can count the number of symbols in a sign-value notation; utilizing this strategy to compare III vs. XX in Roman notation, one would produce an erroneous solution, stating that III is larger than XX, as the first number includes three symbols, while the second number includes only two. Notably, these alternative incorrect strategies give a correct answer for most of the specific number pairs. For example, applying the above mentioned number of symbols, strategy would state that Roman XXX is larger than XX, which in fact is true, although not because XXX includes more digits than XX; rather, the sum of the values in XXX give a larger number than the values of X plus X.

We identified seven strategies that could offer an alternative solution in the comparison task. Some of them might sound bizarre, but we wanted to be confident that the occasionally confused participants were not using any alternative strategies that could fill the gap made by their uncertainty. Some strategies could be applied in only one notation. The seven strategies were:

1. *Misinterpreting the values of the symbols*: Obviously, if someone forgets the value of a symbol, then number processing will fail.
2. *Counting the number of digits*: In sign-value notation, counting the digits instead of summing them up might result in incorrect processing. Conversely, in place-value notation, this strategy always gives the correct result. For example, in Indo-Arabic notation, a three-digit number is always larger than a two-digit number, independent of the specific values.
3. *Summing the values of the digits*: In place-value notation, summing up the values of the digits instead of first multiplying them with their place value gives an incorrect interpretation of that number. However, in sign-value notation, this method is the correct interpretation of the number. In other words, it is an error to handle a place-value number as a sign-value number.
4. *Number of types of symbols*: Counting the types of digits might give an incorrect result; for example, Indo-Arabic 333 vs. 24 could be interpreted as 333 being smaller, as it only includes the symbol 3, while 24 includes 2 and 4. This strategy can be applied in both sign-value and place-value notation.
5. *Decision not based on the largest differing power*: In a multi-power comparison, the largest differing power will specify which number is the larger; therefore, smaller powers are not relevant. However, one might choose a power arbitrarily and make a decision based on that power. For example, comparing Indo-Arabic 24 vs. 53, one might think that, as 4 is larger than 3, 24 is more than 53. This strategy can be used in both sign-value and place-value notations.
6. *The smaller number might be shifted in position when it includes leading zeros*: In place-value notation, if the two numbers have different numbers of powers, one might incorrectly shift the position of the smaller number and make a decision based on this wrong interpretation. For example, in the case of Indo-Arabic 231 vs. 42, one might find that the largest powers are 2 in 231, and 4 in 42, thus making 42 the bigger number. In other words, this solution shifts number 42 to 420. This error is irrelevant in sign-value notation, as the position of the symbols is not informative: (10) + (1) is the same as (1) + (10). Considering this strategy not as shifting position but as shifting power, the strategy is quite improbable in sign-value notation, as shifting powers requires changing all digits; for example, Roman number XXIII should be changed to CCXXX.
7. *Interpreting numbers in base 10*: Finally, we considered a seventh possible problem, which actually cannot be tested in a comparison task. One might think that the numbers are not denoted in base 4, but in base 10. Actually, in a comparison task, interpreting numbers in base 10 will give exactly the same result as interpreting them in base 4.

As mentioned above, in many cases these incorrect strategies will give the correct solution; thus, only the number pairs in which a specific incorrect strategy gives an erroneous result are informative. It would be appealing to find number pair stimuli in which only one incorrect strategy would propose the wrong solution, and the correct strategy, along with all the other wrong strategies, would give the correct answer, as failing these trials would reveal that the specific wrong strategy was used. Unfortunately, this is impossible in some cases. For example, in sign-value notation, when the number of digits would suggest a wrong solution (Strategy 2), the utilization of the “not largest differing power” strategy (Strategy 5) also offers an incorrect response. There are no cases where Strategy 2 gives the wrong solution and Strategy 5 gives the correct result. Thus, whenever it was not possible to choose a number pair where only a single incorrect strategy would propose the wrong answer, we chose an “overlapping” number pair stimulus, in which only two wrong strategies support the wrong result: one is the critical one that we wish to test, and the other is a strategy that could be tested by itself (i.e., there are other number pairs in which the latter incorrect strategy is the only strategy that gives an incorrect result).

#### Participants

Thirty Hungarian undergraduate students from Eötvös Loránd University participated in the study for partial course credit. All participants had normal or corrected to normal vision. The data of 24 subjects were analyzed (two males, age range from 20 to 27) after excluding six participants with a higher than 50% error rates in any of the tested incorrect strategies (see incorrect strategies above and the procedure below). These participants were excluded to ensure that all of the remaining participants understood the structure and logic of both the sign-value and place-value notations. Among the excluded participants, five subjects applied a strategy that counted the number of symbols instead of adding up their values in sign-value notation. Another participant added up the values of the digits in place-value notation (i.e., the place-value numbers were handled as if they were sign-value numbers).

#### Stimuli and procedure

In a comparison task, two multi-power numbers were visible on the left and right sides of the screen, and participants chose the larger number (see Figure 1). In one condition, the numbers were constructed in sign-value notation, while in the other condition, place-value structure was applied.

The presented numbers were between 1 and 63; i.e., numbers that have a maximum of three powers in a base 4 system. The numbers were presented in white against black background. The stimuli were visible until the response button was pressed. A blank screen appeared for 500 ms between trials.

The tasks were presented in two blocks: sign-value and place-value notation blocks. In a block, first, participants learned the symbols; an instruction introduced the new symbols used in the notation. Then, the participants saw one symbol at a time in the middle of the screen and had to press the associated response button. After the response, auditory feedback was given depending on whether the response was correct or not (higher and lower beeps). All new digits were presented twice in a block. The participant performed this practice phase until a block was completed without any error and at least four blocks were accomplished. For most of the participants four blocks of practice was enough to identify the symbols accurately.

Second, the multi-power notation was introduced. To ensure that participants understood that the incorrect strategies described above were erroneous, practice trials tested whether the participants applied these critical strategies. All possible incorrect strategies of the notation were tested with five trials. After each erroneous practice trial, the notation was explained by the experimenter. The trials in the practice phase were randomized.

Finally, participants completed the comparison task. To ensure that participants continued to avoid incorrect strategies, test trials continuously monitored the use of possible wrong strategies while solving the comparison tasks. The incorrect strategies could be verified only with these test trials, as the main trials included numbers that could be solved correctly with any of the incorrect strategies (i.e., all incorrect strategies gave correct solutions to the main trials). An incorrect strategy was followed if more than two errors out of five trials were made in that strategy type.

Former studies on multi-digit Indo-Arabic number comparison have found that (a) the number of digits are used as a shortcut for a decision (i.e., longer numbers are always larger in a place-value system; Hinrichs et al., 1982), and (b) multi-digit comparison is solved with a power-by-power comparison, starting with the largest power (Hinrichs et al., 1982; Poltrock and Schwartz, 1984). To explore the presence of these comparison processes with the present artificial notation, another independent variable included number difference type, with five conditions (Table 3): (a) three-power vs. two-power numbers, e.g., (2)(1)(3) vs. (1)(3) in place-value notation; (b) three-power vs. one-power numbers, e.g., (2)(1)(3) vs. (3); (c) three-power numbers with difference on 16s, e.g., (2)(1)(3) vs. (3)(1)(3); (d) with difference on 4s, e.g., (2)(1)(3) vs. (2)(0)(3); and (e) with difference on 1s, (2)(1)(3) vs. (2)(1)(2).

**Table 3:** **Example stimuli in the two notations (sign-value and place-value numbers) and in number difference types**.

	Sign-value number	Place-value numbers
One leading zero	ҸЯЯƟƟƟ ЯЯƟƟƟ	ĐИ ƟĐИ
Two leading zeros	ƟƟƟ ҸЯЯƟƟƟ	ƟĐИ И
Difference in 16s	ҸЯЯƟƟƟ ҸҸЯЯƟƟƟ	ƟĐИ ĐĐИ
Difference in 4s	ҸЯƟƟƟ ҸЯЯƟƟƟ	ƟŁИ ƟĐИ
Difference in 1s	ҸЯЯƟƟ ҸЯЯƟƟƟ	ƟĐИ ƟĐƟ

The full factorial within-subjects design included notation (sign-value and place-value) and the number difference type with five levels, as described above. Each cell of the design included 15 trials. The stimuli were presented in two notation blocks and the order of the notation was counterbalanced across subjects. In a block, the order of the trials with the number difference conditions and the incorrect strategy trials were randomized. The specific number pairs presented were generated online with the appropriate constrains of the conditions: all stimuli were chosen randomly from the set of number pairs that satisfy the appropriate constrains. Presentation of the stimuli and measurement of RT were managed by PsychoPy software, version 1.61 (Peirce, 2007).

### Results and discussion

**Figure 2:**
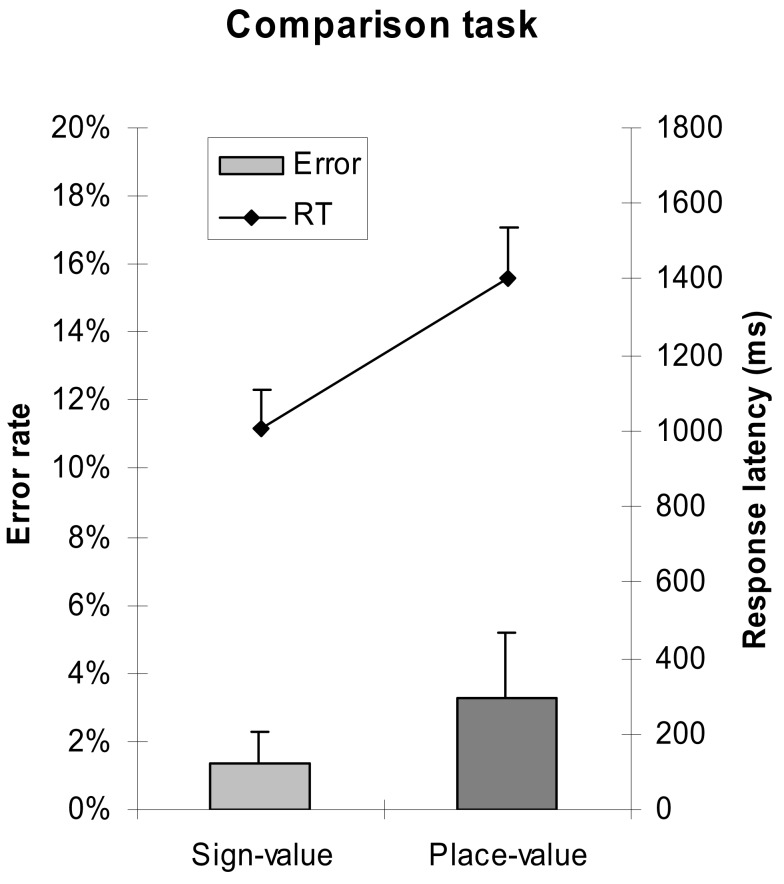
**Error rates and response latencies as a function of number notation in the comparison task**. Error bars represent confidence interval (95%).

Error rates and response latencies were analyzed with a 2 (notation: sign- vs. place-value) × 5 (number difference: one leading zero vs. two leading zeros vs. difference in 16s vs. in 4s vs. in 1s) × 2 (order of notation: sign-value notation first vs. place-value notation first) ANOVA with notation and number difference as within-subjects and order of notation as between-subjects factor. Order of notation neither had a main effect nor interacted with any other factors. We found that comparison in place-value notation was more erroneous and slower than comparison in sign-value notation; *F*(1,22) = 19.774, MSE = 0.001, *p* < 0.001 and *F*(1,22) = 45.393, MSE = 207,517, *p* < 0.001, respectively (see Figure 2 for the notation main effects). This result contrasts with the frequently articulated advantage of the place-value system.

**Figure 3:**
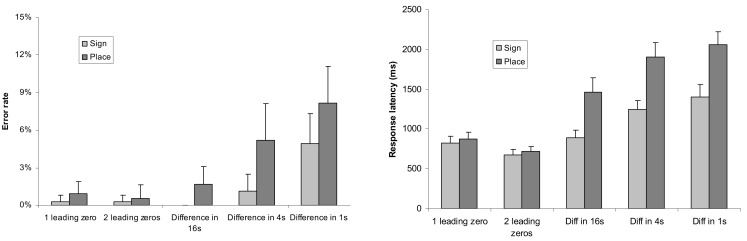
**Error rates (left) and response latencies (right) in the two notations as a function of difference between the two numbers**. Error bars represent confidence interval (95%).

Further analysis explored the presence of comparison strategies formerly observed in multi-digit Indo-Arabic comparison tasks (Hinrichs et al., 1982; Poltrock and Schwartz, 1984). The previous ANOVA on error rates revealed a main effect of number difference; *F*(4,88) = 19.511, MSE = 0.0023 *p* < 0.001. The interaction between the number difference and notation factors was not significant. The previous ANOVA on response latencies showed a main effect of number difference; *F*(4,88) = 179.876, MSE = 53,081, *p* < 0.001. The interaction of the number difference and notation factors also proved to be significant; *F*(4, 88) = 36.988, MSE = 34,024, *p* < 0.001: in leading zero conditions the response latencies did not differ between the two notations (Figure 3).

These behavioral data are consistent with the formerly known multi-digit Indo-Arabic number processing. First, response latencies show a relatively fast solution for trials with leading zeros (Hinrichs et al., 1982). Similarly, in the present data, if the largest powers of the two numerals differ, then the number with the larger power is quickly chosen (see the lower error rate and faster reaction time in the leading zero conditions in Figure 3). In place-value numbers, the longer number is the larger, and the decision might be made by simple perceptual procedure, without processing the numbers. In sign-value notation, although the length of the stimuli correlates with the leading zeros, such an unambiguous relation is not present. Thus, in sign-value notation the first symbols of the two numbers should be compared. Second, in the three-power number pairs, the larger the differing power is, the faster the comparison is (see the increasing error rate and response latency in the difference in 16s, 4s, and 1s conditions in Figure 3). This result might reflect a distance effect (Dehaene et al., 1990), or it can also be interpreted that participants started the comparison with the largest power and continued it until the difference between the two numbers was found (Poltrock and Schwartz, 1984). This serial decomposing interpretation is strengthened by the fact that more than two-digit Indo-Arabic comparison might be processed serially, as non-serial holistic or parallel processing of the powers is plausible only with familiar numbers, such as frequently observed two-digit numbers (Dehaene et al., 1990; Verguts and de Moor, 2005; but see also Korvorst and Damian, 2008). Furthermore, children who have fewer experiences with multi-digit numbers tend to perform comparisons serially (Nuerk et al., 2004), which also strengthens the serial processing hypothesis.

**Figure 4:**
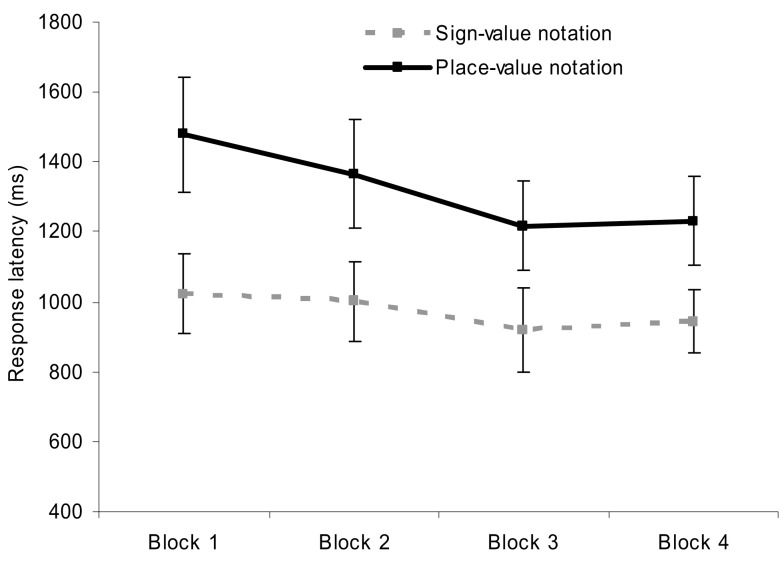
**Response latencies in the two notations as a function of time**. Error bars represent confidence interval (95%).

To investigate the performance improvement over time, the trials were grouped into four blocks. A 2 (notation) × 4 (blocks) repeated measures ANOVA on error rates revealed only a main effect of notation, but not a main effect of blocks or interaction. A similar 2 × 4 ANOVA on the response latencies (Figure 4) showed a main effect of notation, a main effect of blocks, *F*(3,69) = 6.12, MSE = 56,889, *p* = 0.001, but no interaction.

## Experiment 2

The comparison tasks, in a strict sense, could have been solved by ordering the symbols without considering their values. To rule out this potential problem, we utilized the more complex task of addition. Moreover, in the comparison task, participants might have misunderstood the base 4 system and could have interpreted the multi-power numbers as base 10 numbers, as both interpretations implied the same solution. However, in the addition task, misunderstanding the base would cause a carry error, for example, 2 + 3 is 5 in a base 10 system, but the result is (1)(1) in a base 4 notation. Third, addition was also a vital procedure in ancient times, as complex notations were frequently applied for addition, such as for summing assets or taxes (Ifrah, 1999).

### Methods

#### Participants

Twenty-two Hungarian undergraduate students from Eötvös Loránd University participated in the study for partial course credit. Nineteen subjects were analyzed (four males, age range from 20 to 23) after excluding participants with more than 20% overall error rate. In contrast with the comparison task, no incorrect strategies test trials were needed in the addition task, as the aforementioned incorrect strategies would result in wrong solutions in the addition tasks. Consequently, exclusion of the participants was not based on incorrect strategy test trials, but on the overall performance in the addition task.

#### Stimuli and procedure

**Figure 5:**
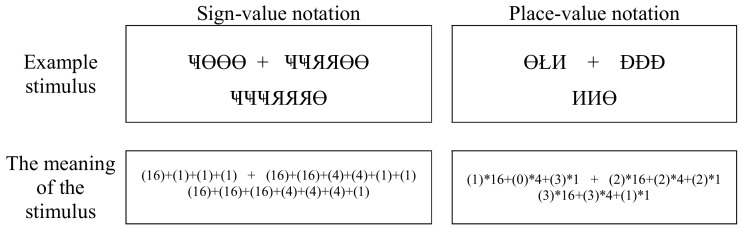
**Example of sign-value (left) and place-value (right) stimuli in the addition task**. Both examples show the addition of 19 + 42, with the correct result of 61. The bottom row shows the correct interpretations of the numbers. Numbers in parentheses represent the symbols. Note that participants did not have to transcode these base 4 numbers to a base 10 place-value notation; i.e., to Indo-Arabic notation to solve the task.

The same artificial notational system was applied as in the previous experiment. In a trial, a multi-power addition was visible in the middle of the screen, and a proposed solution appeared at the bottom of the screen (see Figure 5). Participants decided whether the proposed result was correct or not. Stimuli were visible until the response button was pressed. Between the trials, a blank screen appeared for 500 ms. Both the operands and the results were three-power numbers larger than 15; no leading zeros were included.

To compare the present artificial number-learning paradigm with the formerly known multi-digit Indo-Arabic addition, two manipulations of the stimuli were applied. To investigate serial power-by-power processing (Deschuyteneer et al., 2005), the incorrect result could differ in the 1s [e.g., (3)(2)(3) vs. (3)(2)(0) in a place-value notation], in the 4s [e.g., (3)(2)(3) vs. (3)(1)(3)], and in the 16s [e.g., (3)(2)(3) vs. (1)(2)(3)]. Second, to test whether performance of a task with carry is worse than without carry (Deschuyteneer et al., 2005), the trials included no carrying [e.g., (2)(1)(2) + (1)(0)(1)], one carry [e.g., (2)(1)(2) + (1)(0)(3)], or two carries [e.g., (2)(1)(1) + (1)(2)(3)].

The tasks were presented in two blocks: sign-value and place-value notation. In each block, participants first learned the new symbols with the same procedure as in the first experiment, then practiced the addition in the new notation. To ensure that participants understood the task, the following types of additions were practiced: (1) in sign-value notation, two one-digit addends; (2) in place-value notation, two one-digit addends with no carry; (3) in both notations, multi-power addition without carry; (4) in both notations, multi-power addition with one carry; and (5) with two carries. After each erroneous practice trial, the rules of addition were explained by the experimenter.

In the main part of the experiment, half of the trials showed a correct result, and the other half showed an incorrect sum. In a notation block, 120 trials were presented. In both notations, a factorial design included erroneous power and carry factors, with 20 trials in each erroneous condition, 60 trials in the correct result condition, and 30 trials in each carrying condition (see the detailed distribution of the trials in the design cells in Table 4). The order of the notation block was counterbalanced across subjects. In a notation block, the order of the trials was randomized.

**Table 4:** **Number of trials in the cells of the design within a specific notation**.

	Erroneous result			Correct result
	Error on 16s	Error on 4s	Error on 1s	No error
No carry-over	5	5	5	15
Carry-over from 1s	5	5	5	15
Carry-over from 4s	5	5	5	15
Carry-over from 1 to 4s	5	5	5	15

### Results and discussion

**Figure 6:**
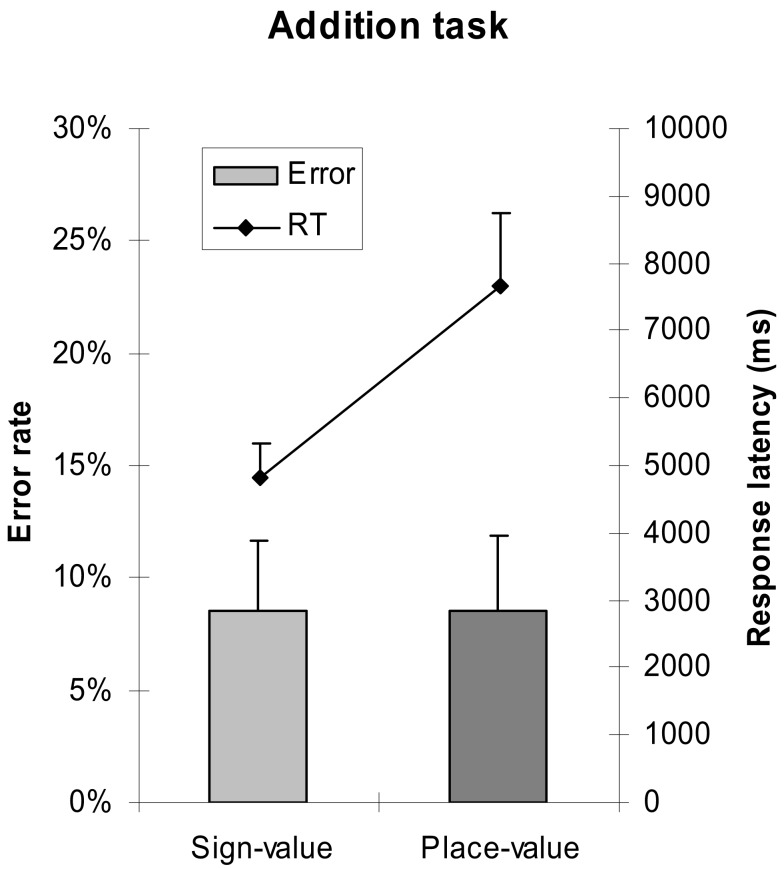
**Error rates and response latencies as a function of number notation in addition task**. Error bars represent confidence interval (95%).

A 2 (notation: sign-value vs. place-value notation) × 4 (erroneous power: no error vs. error in 16s vs. error in 4s vs. error in 1s) × 4 (carry: no carries vs. carry from 1s vs. carry from 4s vs. carry from 1s to 4s) repeated measures ANOVA was applied to analyze error rates and median response latencies of correct responses. We found that, consistent with the comparison task, addition with place-value notation was slower than addition with sign-value notation [*F*(1,18) = 47.787, MSE = 23,833,720, *p* < 0.001], while the error rate did not differ significantly between the two notations (Figure 6).

**Figure 7:**
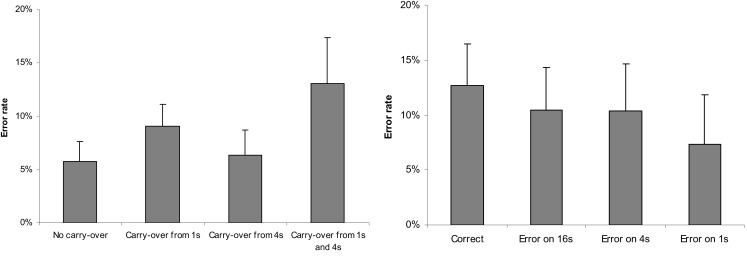
**Error rates as a function of carry (left) and as a function of erroneous power (right)**. Error bars represent confidence interval (95%).

Further analysis explored the effects formerly known from multi-digit Indo-Arabic addition (Deschuyteneer et al., 2005). The previous ANOVA on error rates revealed no effect of notation in the main effect or in the interactions. However, carry and erroneous power main effects were significant, *F*(3,54) = 4.335, MSE = 0.017, *p* = 0.008 and *F*(3,54) = 6.337, MSE = 0.017, *p* = 0.001, respectively (Figure 7). Increasing the number of carries, the task became more difficult and errors on smaller powers of the stimuli resulted in fewer erroneous responses. Moreover, carry × erroneous power and notation × carry × erroneous power interactions also proved to be significant; *F*(9,162) = 2.55, MSE = 0.016, *p* = 0.009 and *F*(9,162) = 2.189, MSE = 0.013, *p* = 0.025, respectively. In the carry × erroneous power interaction, the interaction component of the ANOVA model revealed that, in the case of correct proposed solution, two carries increased the error rate, while the no carry condition decreased the error rate relative to the additions with erroneous proposed results.

**Figure 8:**
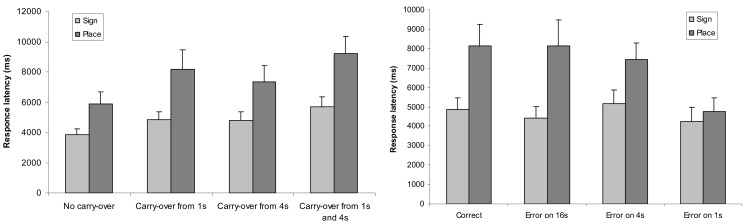
**Response latency in sign-value and place-value notations as a function of carry (left) and as a function of erroneous power (right)**. Error bars represent confidence interval (95%).

The previous ANOVA on response latencies (Figure 8) revealed main effect of notation (as reported above), main effect of carry, and main effect of erroneous power; *F*(3,54) = 47.272, MSE = 3,458,416, *p* < 0.001 and *F*(3,54) = 27.032, MSE = 6,041,738, *p* < 0.001, respectively. Furthermore, all interactions became significant: notation × carry, *F*(3,54) = 5.605, MSE = 2,723,516, *p* = 0.002; notation × erroneous power, *F*(3,54) = 14.912, MSE = 6,522,541, *p* < 0.001; carry × erroneous power, *F*(9.162) = 2,342, MSE = 2,570,820, *p* = 0.017; and notation × carry × erroneous power, *F*(9,162) = 2.117, MSE = 1,765,930, *p* = 0.031. While carry and erroneous power modify the notation effect, reflected in the interactions, none of these factors reverse the notation effect.

This more detailed analysis reflects a similar processing in the addition task than was observed previously with multi-digit Indo-Arabic notation (Deschuyteneer et al., 2005). First, the more carries the addition included, the more difficult they proved to be. Second, participants started addition with 1s and continued to higher powers until they found an error; otherwise, they responded that the addition was correct. The more steps the problem solver made until the response, the more likely it was that they made an error and the more time was required. Consequently, the easiest tasks were the trials with the erroneous power in the 1s, while erroneous 4s and 16s were more difficult. The correct additions were as difficult as the trials with error in the 16s, reflecting that, after checking all powers, participants decided that the proposed addition was correct. The reason to use this small-power-first strategy is to put a relatively low load on working memory while computing carries (Deschuyteneer et al., 2005). A carry would change the value of the neighboring larger power, and starting addition from a large power occasionally would cause modification of an already computed result. However, in the sign-value notation, a different strategy is visible: the trials with errors in the 4s were slower than the trials with errors in the 1s or in the 16s, suggesting that a computationally less efficient strategy was also applied, in which the processing started from the largest power (16s) to the smallest one. This phenomenon might have some root in the perceptual nature of sign-value notation, although its exact nature is not yet clarified.

To test the effect of the order of presentation a 2 (notation) × 2 (notation order) ANOVA was run with notation as a within-subject and order of notation as a between-subject factor. The ANOVA on error rates did not show a main effect neither for the notation nor for the order of the notation, while the interaction was significant, *F*(1,17) = 7.435, MSE = 0.005, *p* = 0.014. When sign-value notation was learned first, error rate with sign-value notation was lower (7.3%) than with place-value notation (10%). In contrast, when place-value notation was learned first, error rate with sign-value notation was higher (9.5%) than with place-value notation (7.4%). Thus, the notation learned first showed lower error rate. The same ANOVA on response latency revealed a main effect of notation, *F*(1,17) = 35.568, MSE = 67,315,998, *p* < 0.001, while neither the main effect for notation order, nor the interaction was significant.

**Figure 9:**
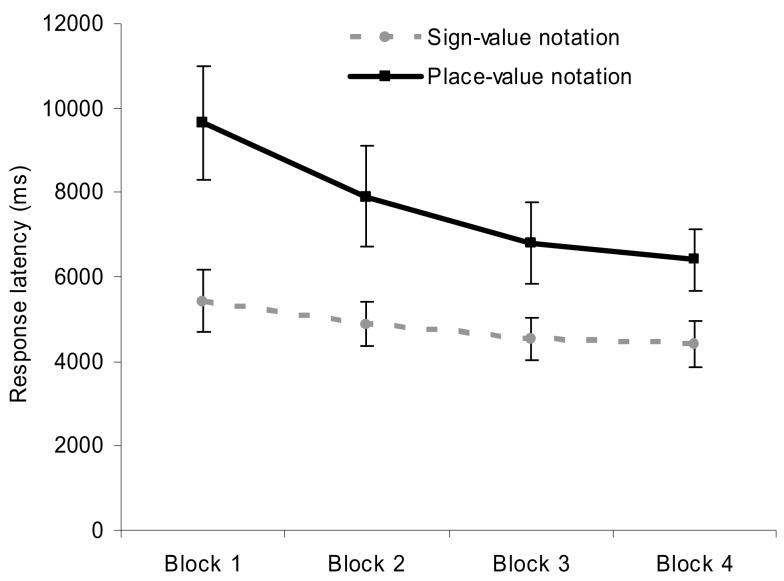
**Response latencies in the two notations as a function of time**. Error bars represent confidence interval (95%).

To investigate the performance improvement over time, trials were grouped into four blocks (see Figure 9). In a 2 (notation) × 4 (blocks) repeated measures ANOVA on error rates no significant effect was observable. A similar 2 × 4 ANOVA on response latencies showed a main effect of notation, a main effect of blocks, *F*(3,54) = 32.22, MSE = 1,084,409, *p* < 0.001, and a significant interaction, *F*(3,54) = 9.17, MSE = 1,043,334, *p* < 0.001. The interaction reflected a stronger improvement in place-value notation than in sign-value notation.

## Experiment 3

Adults can compare and add numbers better in sign-value notation than in place-value notation. One possible explanation for this phenomenon is that adults have extensive experience with place-value Indo-Arabic numerals, and this former knowledge about a place-value system could cause interference in the newly acquired place-value system, while the sign-value system is untouched by such an influence. To investigate this possibility, the comparison task was solved by preschool children who have less experience with the place-value system and their performance shows higher variance.

The previous results could have been attributed to the specific symbol-value assignments. For example, the symbol Ɵ meant 1, although the symbol itself may remind some participants of the digit 0. This interference might cause an artifact, and the results described above could be attributed to this uncontrolled factor. To control for this possible artifact the symbol-value assignment was randomized for every participant. If the previous results are the consequence of a specific symbol-value assignment artifact, then the effects should disappear or at least diminish in the random symbol-value assignment version.

### Methods

#### Participants

Forty-five preschool children participated in the study; 24 girls and 21 boys; mean age 6–5, range 5–8 to 7–5.

#### Stimuli and procedure

The same comparison task was used as in Experiment 1, with the following modifications. Each cell of the design included 10 trials instead of 15 trials, to shorten the length of the experiment. The same practice trials that were applied in the adult version were repeated twice to support the learning process. The same symbols were used as in Experiment 1; however, the values of those symbols were randomized for every participant. For example, the symbol Ɵ could mean 1 for some participants, 2 for some others, etc.

Understanding the new artificial notation could be influenced by former knowledge of other place-value and sign-value number notations. To control for the effect of the already learned Indo-Arabic and Roman numbers, a number reading task was given. Children had to read (a) single-digit Indo-Arabic, (b) multi-digit Indo-Arabic, and (c) Roman numbers. The numbers were presented in the middle of the screen, and children read the number out load. In the single-digit Indo-Arabic task all digits from 1 to 9 (0 excluded) were presented. In the multi-digit Indo-Arabic task, 10 numbers from 11 to 29 were presented; five even and five odd numbers, and five numbers from the teens and five from the twenties. In the Roman number task, all numbers from 1 to 9 were presented.

### Results and discussion

Understanding of the notations was measured with the error rates in the incorrect strategy test trials (see the methods in Experiment 1). An incorrect strategy was successfully avoided by a child if the error rate in that strategy was less than 50%; i.e., a maximum of two errors were made out of the five trials. Learning the new number notation in a single session was partially successful for preschool children: 25 (56%) understood sign-value notation and 14 (31%) could apply place-value notation appropriately in the task, resulting in nine children (20%) who could learn both notations. The difference between the learning performance of the two notations was significant [χ^2^(1, *N* = 45) = 4.762, *p* = 0.029). As in the case of adults, in Experiment 1, the most frequent error sources were that (a) children summarized the value of the digits in place-value notation (i.e., place-value notations were handled as sign-value notation; 25 children (56%) used this erroneous strategy); and (b) they counted the number of the digits instead of summarizing them in sign-value notation [17 children (38%) applied this incorrect solution].

To control for former experiences with written number notations, knowledge about single- and multi-digit Indo-Arabic and Roman numbers were measured. Preschool children were confident with the Indo-Arabic numerals, making only 4% errors on average in reading single-digit Indo-Arabic numbers, and making 22% errors with multi-digit Indo-Arabic numbers. Roman numbers were not known by these children, as reflected in the 85% error rate. While preschool children have definitely less experience with Indo-Arabic numbers than adults, they still have some knowledge about the multi-digit place-value notation. Therefore it is possible that this cultural influence might already have an effect on the artificial number notation paradigm. To test this hypothesis, the error rate of multi-digit Indo-Arabic number reading was correlated with the error rate of both the sign-value and the place-value comparison tasks. If knowledge about multi-digit Indo-Arabic numbers causes bias on the new number notation learning, then a positive correlation with place-value and a negative correlation with sign-value notation is expected. Multi-digit Indo-Arabic number reading error rates correlated positively with both sign-value comparison error rate, *r*(45) = 0.356, *p* = 0.017 and place-value comparison error rate, *r*(42) = 0.307, *p* = 0.048. The two correlations did not differ significantly, *Z* = −0.3, *p* = 0.76. These results reveal that experience with Indo-Arabic numbers measured as multi-digit reading did not interfere with the learning of the new artificial number systems.

#### Additional analysis

For further analysis, only the data of the children who understood both sign-value and place-value notations, verified by the incorrect strategy test trials, were used. The remaining nine children were two girls and seven boys, mean age 6–8, range 6–1 to 7–4. Although the children who understood both notations were somewhat older than those who had difficulties with them (6–8 vs. 6–5 years), the difference was not significant, *t*(43) = −1.329.

**Figure 10:**
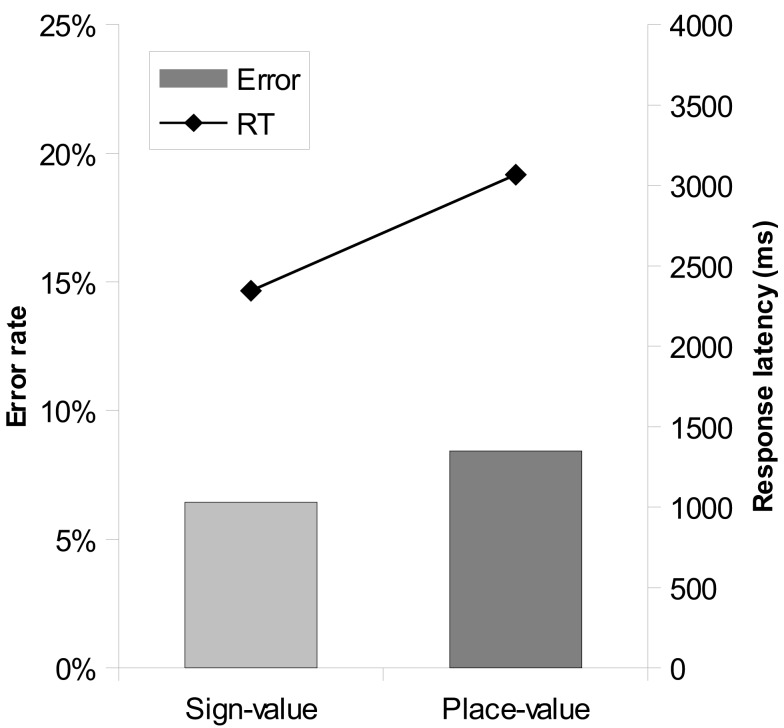
**Error rates and response latencies as a function of number notation in comparison task**. Error bars represent confidence interval (95%).

Error rates and median response latencies of the correct responses were analyzed with a 2 (notation: sign- vs. place-value) × 5 (number difference: one leading zero vs. two leading zeros vs. difference in 16s vs. in 4s vs. in 1s) repeated measures ANOVA. While error rate did not differ between the two notations, place-value notation comparisons were slower to solve than sign-value tasks with marginal significance, *F*(1,8) = 0.679, MSE = 0.015, *p* = 0.434 for error rate and *F*(1,8) = 4.182, MSE = 1,062,876, *p* = 0.075 for RT (Figure 10).

**Figure 11:**
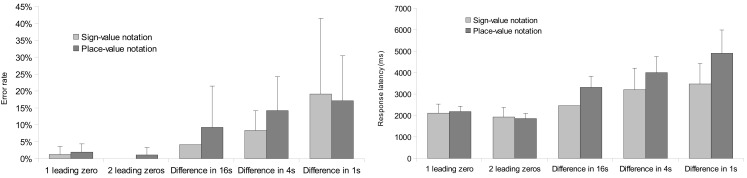
**Error rates (left) and response latencies (right) in the two notations as a function of difference between the two numbers**. Error bars represent confidence interval (95%).

As in Experiment 1, the comparison strategies were also investigated (Figure 11). A 2 (notation: sign- vs. place-value) × 5 (number difference: one leading zero vs. two leading zeros vs. difference in 16s vs. in 4s vs. in 1s) repeated measures ANOVA on error rates revealed a main effect of number difference, with Huynh–Feldt correction, *F*(4,32) = 4.043, MSE = 0.055, *p* = 0.047, while the interaction was not significant. The response latencies in a similar ANOVA showed a main effect of number difference; *F*(4,32) = 21.958, MSE = 765,188, *p* < 0.001. The interaction of the two factors was also significant; *F*(4, 32) = 4.64, MSE = 379,267, *p* = 0.05: in the leading zero conditions the notation effect is missing.

The error rate and response latency patterns reflect the same strategies that were observed in adults in Experiment 1 and in former studies (Hinrichs et al., 1982; Poltrock and Schwartz, 1984): (a) comparisons with leading zeros are processed faster, and (b) multi-power comparison starts with the largest power and continues with the smaller ones until a difference in the numbers is found.

The specific symbol-value assignment was randomized in this experiment to control for the effect of specific symbols that might cause interference with other known symbols. The pattern of the errors and response latencies are basically the same as found in Experiment 1, suggesting that the effects described above can not be attributed to the specific symbols and their assignments utilized in our study.

To summarize, for preschool children, sign-value notation is easier to use than place-value notation in multi-power comparison tasks. This result did not depend on former experience with other written number notations. Finally, preschool children used the same strategies adults did.

## Experiment 4

It is possible that the notation effect is based on some non-essential properties of the notation system, rather than on the logic of the notation. Since the number notations display the powers in an ordered sequence (i.e., largest powers are on the left), in the case of sign-value notation it is not necessary to learn the symbols as strictly as in place-value notation, because the position of the symbol also gives information about the value of the symbol (e.g., the leftmost symbols should be 16s). This feature of the sign-value notation might contribute to the advantage of sign-value notation, or according to a more extreme scenario, it is possible that the whole notation effect is simply an artifact of the position information in sign-value notation.

Additionally, in the previous experiments base 4 systems with 3 powers were used. This means that while participants had to learn three symbols in sign-value notation, they had to learn four symbols in place-value notation. Again, sign-value notations had an advantage over place-value notations in the previous experiments.

To test the possible effect of position information and amount of symbols to learn on notation effect, we designed a follow-up experiment in which position information and the number of symbols were controlled.

### Methods

#### Participants

Eighteen Hungarian undergraduate students from Eötvös Loránd University participated in the study for partial course credit. All participants had normal or corrected to normal vision. The data of 16 subjects were analyzed (two males, age range from 19 to 26) after excluding two participants with a higher than 50% error rates in any of the tested incorrect strategies (see incorrect strategies above and the procedure below).

#### Stimuli and procedure

The same stimuli were used and the same procedure was followed as in Experiment 1 with the following modifications.

To control for the number of symbols to be learned even more strictly, base 3 number system (instead of base 4 in the previous experiments) with three-power numbers were utilized. In this base 3 system, the powers could be 1, 3, and 9. Thus, the same number of symbols should be learned both in the sign-value notation (symbols for 1, 3, and 9) and in the place-value notation (symbols for 0, 1, and 2). The assignment of symbols and values were randomized as in Experiment 3 to ensure that the notation effect is not the result of some specific symbol processing.

To test the effect of position information in the sign-value notation, the position information should be removed. In the sign-value notation the position information cannot be used when some of the powers are missing in a number (which would be denoted by zeros in a place-value notation), thus, for example after 9s the participants cannot be sure whether the next symbols denote 3 or 1. Leading zeros should not be used as test stimuli, because leading zeros in place-value notation are processed by length shortcut as revealed in the first experiment. Thus, test number pairs had a x0x and xx0 power structure, in which x could be any non-zero power, and the largest powers were equal, so thus the task could be solved only on the medium power. In these tasks participants cannot rely on position information, because in the two numbers after the 9s different symbols show up, and to compare the powers, the participant must recall the value of that digit.

The new test condition was added to the five conditions applied in Experiment 1: (a) one leading zero, (b) two leading zeros, (c) difference in 9s, (d) difference in 3s, and (e) difference in 1s.

### Results and discussion

**Figure 12:**
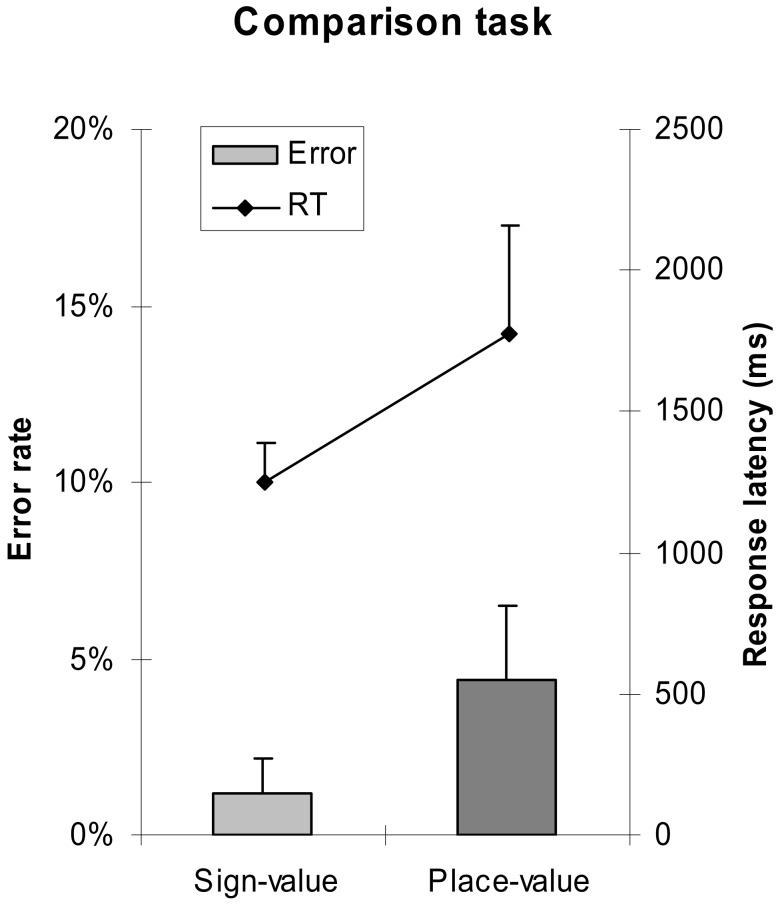
**Error rates and response latencies as a function of number notation**. Error bars represent confidence interval (95%).

**Figure 13:**
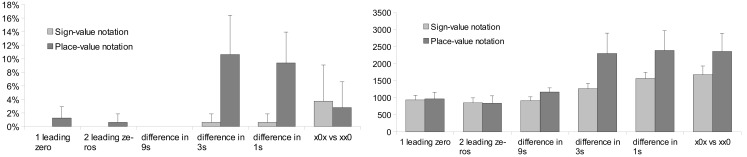
**Error rates (left) and response latencies (right) in the two notations as a function of difference between the two numbers**. Error bars represent confidence interval (95%).

Error rates and median response latencies of the correct responses were analyzed with a 2 (notation: sign- vs. place-value) × 6 (number difference: one leading zero vs. two leading zeros vs. difference in 16s vs. in 4s vs. in 1s vs. the new test condition, x0x and xx0 pairs) × 2 (order of notation: sign-value notation first vs. place-value notation first) ANOVA with notation and number difference as within-subject and order of notation as between-subject factors (see Figure 12 for the notation main effect; Figure 13 for the notation × number difference interaction). In the error rates both notation and power condition factors were significant, *F*(1,14) = 17.43, MSE = 0.06, *p* = 0.001 and *F*(5,70) = 4.512, MSE = 0.019, *p* = 0.001, respectively. Place-value notation caused more errors (4.3%) than sign-value notation (0.6%). According to the *post hoc* LSD tests the difference in 3s, difference in 1s were harder to solve than the difference in 9s and leading zeros condition. The interaction of the two factors were also significant, *F*(5,70) = 6.09, MSE = 0.015, *p* < 0.001, in which case the place-value notation showed higher error rates than sign-value notation in the difference in 3s and difference in 1s conditions. A planned *t*-test in the new test condition did not show a significant difference between the two notations. In the response time analysis both main effects and the interaction was significant, *F*(1,14) = 10.82, MSE = 11,222,159, *p* = 0.005 in the notation factor, *F*(5,70) = 56.95, MSE = 9,434,888, *p* < 0.001 in the power condition factor, and *F*(5,70) = 8,79, MSE = 1,542,484, *p* < 0.001 in the interaction. A planned *t*-test investigated the notation effect in the new test condition. The difference in the “x0x and xx0” condition was significant, *t*(15) = 2.78, *p* = 0.014. The order of the notation was not significant neither in error rates, nor in response latency, and it did not interact with any other factors.

The notation effect did not disappear in the “x0x and xx0,” in which the position information was unavailable for the participants in the sign-value notation. Thus, it is improbable that the notation effect would be solely the artifact of this extra position information in sign-value notation. To summarize, stricter control for the number of symbols to learn and the position information in the sign-value notation does not remove the notation effect.

## General Discussion

In the present study, participants could more easily compare and add new base 4 (or base 3) artificial multi-power numbers in sign-value notation than in place-value notation, the result of which works against the conventional view of the overall superiority of place-value system. This finding cannot be the result of former experience with place-value Indo-Arabic system causing interference with the new artificial notations, since preschool children showed the same notation effect in the comparison task independently of their former Indo-Arabic number experiences. The relative advantage of sign-value notation is consistent with the popularity of sign-value notation in the history of culture (Ifrah, 1999; Chrisomalis, 2010) and with the proposed simple computation algorithms (Anderson, 1956; Lazarides, 1970; Detlefsen et al., 1976; Kennedy, 1981; Schlimm and Neth, 2008). These results highlight the essential role of number notation in numerical processing.

Why was sign-value notation easier to process than place-value notation in simple numerical tasks? Representing multi-power numbers requires a special representation, although its nature is debated. According to former models, this representation is assumed to be in a verbal form (Dehaene et al., 1999; Spelke and Tsivkin, 2001), in an Arabic visual form (Dehaene, 1992), or in an abstract structure (McCloskey, 1992). How can these models explain the notation effect? The Arabic visual number form cannot account for the results (Dehaene, 1992; Dehaene et al., 1993), as this model utilizes a place-value representation that should have predicted a faster place-value notation processing, which was not the case. Second, the notation effect might be also incompatible with verbal number representation. Verbal representation stores numbers in the form of number words, and number words are neither sign-value nor place-value notations; rather, they are distinctive forms of notations. Importantly, there are no particular considerations suggesting a preference for translating any of the notations used here to verbal representation. Third, the abstract representation stores both the power and the number of powers in a symbolic form, which does not favor any of the notations used here.

**Figure 14:**
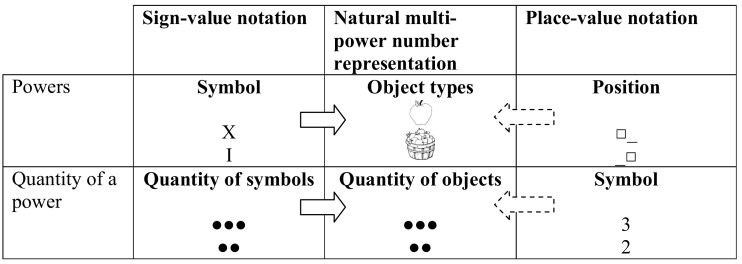
**Hypothetical translation processes from sign-value and place-value notations to natural multi-power number representation**.

Because none of the three former number representation models accounts for the notation effect, we propose here an alternative model, termed the natural multi-power number representation, based on the numerical representation of objects and groups. This number representation might represent numbers as specific number of objects, in which some type of objects may represent items, while other types of objects can represent groups or higher powers (see the middle column in Figure 14). We suggest that sign-value systems can be processed more easily than place-value notation because sign-value notation has a more similar structure to this representation, and it might be transcoded more easily to this natural multi-power number representation (Figure 14). The similarity can be captured in at least four aspects. First, in sign-value notation, comprehending a value in a selected power is performed by quantifying the symbols, analogous to the object quantification. In contrast, place-value denotes the quantity within a power with symbols (second row of Figure 14). Second, in sign-value notation, the powers are denoted by symbols which can be more directly interpreted as a unit or a group (power), compared to the place-value representation, in which the power is denoted by the position (first row of Figure 14). Third, in the addition task, addition within a specific power resembles the placing of objects next to each other (e.g., Ɵ + ƟƟ = ƟƟƟ), while place-value addition is more abstract: adding a symbol to another symbol results in a third arbitrary symbol (e.g., Ł + Đ = Đ, meaning 1 + 2 = 3, as in the previous example, although the meaning of this notation is less transparent). Fourth, in sign-value addition, handling carries is like exchanging some symbols for a “larger” symbol, which is the meaning of that “larger” symbol (e.g., exchanging five Is for a V in Roman notation), while in place-value addition, handling carries results in the appearance of a small number (i.e., the number 1) on a larger position. In other words, in place-value notation the meaning of the symbol is mixed with its position.

To summarize, the analogous structure of natural multi-power number representation and sign-value notation might make transcoding fast, while processing the place-value notation with a divergent structure requires more abstraction and causes difficulties. It is important to stress that we do not imply that the abstract code or the triple code model with the Arabic visual number form and verbal number representation would be invalid; rather, we propose a new complementary number representation that can be seen as the base of representing exact multi-power numbers. The hypothesis of natural multi-power number representation cannot be constructed from our results in a strict sense, as the experiment was not designed to explore detailed representational and transcoding issues; rather, the hypothesized representation seems a reasonable possibility in light of the theoretical consideration and in light of our result conflicting with previous assumptions about the superiority of place-value notations.

We highlight that only simple numerical tasks were tested in the present study, and all the presented artificial number systems were base 4 or 3 systems with maximum three powers in use. It is not entirely known how generalizable our results are. Changing the base or the power of the system changes the number of symbols a user should learn and changes the average length of the numbers. Both of these factors might influence the processing speed and the error rates. While in the present study we controlled for these factors to investigate only the effect of the structures of the notations, for example, a base 10 system with values of different order of magnitudes might be processed differently.

The data presented herein might explain why sign-value notation was popular for centuries, even when alternative place-value notation was available (Ifrah, 1999; Chrisomalis, 2010): the sign-value system is easy to learn and apply for numerical tasks that were common in ancient times. Although these results might have resolved one problem, they also raise another one: if the sign-value notation is easy to learn and apply, why do most cultures today use a place-value system, the Indo-Arabic numbers? Indeed, sign-value notations have their drawbacks; many authors emphasize that sign-value numbers are long and thus unmanageable (Cajori, 1924; Menninger, 1992; Zhang and Norman, 1995; Dehaene, 1997; Ifrah, 1999). Furthermore, simulation shows that arithmetical computations might require more steps in sign-value than in place-value notation (Schlimm and Neth, 2008). Several factors might influence the efficiency of the number notations. First, expertise of the user can be essential. The sign-value system might be applied more rapidly by novices, as in our experiment, or in ancient cultures where the amount of experience with numbers was limited. However long-term learning might reverse the advantages of number notations making sign-value calculation slower for expert users. We suggest that the complexity of the economy and culture in general could reach a specific point where the experience of the individuals with numbers and calculations could be high enough to motivate a change from sign-value notation to a place-value system. Second, the number of symbols a user should learn and the length of the numbers could vary with the base number and the largest power a system expresses. These factors might influence the efficiency of use of a number notation. While in the present study we controlled for many properties of the number notations to ensure the structure of the notations were the only difference between the presented notations, these parameters were not controlled throughout the history of culture. For example, base 10 systems were used while probably applying only a few powers (i.e., rarely using numbers in the order of thousands or larger). Similarly, while most number users could be regarded as novices centuries ago, and only a few experts used numbers frequently enough to switch to advanced strategies, most of us today can be considered experts by the standards of previous centuries. These differences between our carefully controlled experiments and historical conditions leave some questions open as to what specific parameters might reverse the notation effect.

To summarize, our results reveal that if parameters other than the notation itself are controlled, then sign-value notation is easier to apply than place-value notation in simple numerical tasks. We propose that this notation effect can be explained with a natural multi-power number representation based on object representation. These results highlight the elementary role of number notations in number representation and imply that the effects originating from the number representation and the effects originating from the notation of numbers should be distinguished.

## Conflict of Interest Statement

The authors declare that the research was conducted in the absence of any commercial or financial relationships that could be construed as a potential conflict of interest.
